# Young plasma ameliorates aging-related acute brain injury after intracerebral hemorrhage

**DOI:** 10.1042/BSR20190537

**Published:** 2019-05-17

**Authors:** Jun-Jie Yuan, Qin Zhang, Chang-Xiong Gong, Fa-Xiang Wang, Jia-Cheng Huang, Guo-Qiang Yang, Liang Liu, Kai Zhou, Rui Xu, Qiong Chen, Yu Zhou, Xiao-Yi Xiong, Qing-Wu Yang

**Affiliations:** Department of Neurology, Xinqiao Hospital, The Army Medical University, Third Military Medical University, Chongqing 400037, China

**Keywords:** Aging, acute brain injury, intracerebral hemorrhage, IGF-1, young plasma

## Abstract

Aging has been shown to contribute to both the declined biofunctions of aging brain and aggravation of acute brain damage, and the former could be reversed by young plasma. These results suggest that young plasma treatment may also reduce the acute brain damage induced by intracerebral hemorrhage (ICH). In the present study, we first found that the administration of young plasma significantly reduced the mortality and neurological deficit score in aging ICH rodents, which might be due to the decreased brain water content, damaged neural cells, and increased survival neurons around the perihematomal brain tissues. Then, proteomics analysis was used to screen out the potential neuroprotective circulating factors and the results showed that many factors were changed in health human plasma among young, adult, and old population. Among these significantly changed factors, the plasma insulin-like growth factor 1 (IGF-1) level was significantly decreased with age, which was further confirmed both in human and rats detected by ELISA. Additionally, the brain IGF-1 protein level in aging ICH rats was markedly decreased when compared with young rats. Interestingly, the relative decreased brain IGF-1 level was reversed by the treatment of young plasma in aging ICH rats, while the mRNA level was non-significantly changed. Furthermore, the IGF-1 administration significantly ameliorated the acute brain injury in aging ICH rats. These results indicated that young circulating factors, like IGF-1, may enter brain tissues to exert neuroprotective effects, and young plasma may be considered as a novel therapeutic approach for the clinical treatment of aging-related acute brain injury.

## Introduction

Acute brain injury caused by intracerebral hemorrhage (ICH) results in high mortality and morbidity rates, as approximately half of ICH patients die within six months of the hemorrhage and those patients who survive often have permanent brain damage [1,2]. Importantly, advanced age is a predominant risk factor for the development of ICH [3,4], as more severe disabilities occur in elderly stroke patients [5]. These phenomena and results suggest that the aging could significantly aggravate the outcomes of ICH patients.

Aging is a chronic process of progressive functional decline at the cellular, tissue, and organ levels [6]. Recent studies have shown a direct correlation between aging and the decline of normal brain function [7,8], which also contributes to acute brain damage and increased risk of death when associated with active brain injury [5,9]. Intriguingly, heterochronic parabiosis, a technique that links the circulatory systems of an old and young mouse together, and young plasma could rejuvenate several hallmarks of aging and improve the biofunctions in old mice [10–12]. Furthermore, circulating factors have been shown to modulate aging and rejuvenate several organs in the human body, such as the brain, heart, and muscles [10,12–15].

Therefore, we hypothesized that young plasma and its factors may also reduce acute brain injury caused by ICH in aging animals. In the present study, we found that the young plasma can significantly reduce the acute brain injury and mortality of aging rodents after ICH. The circulating insulin-like growth factor 1 (IGF-1) level was markedly decreased with age detected by using the proteomic analysis and ELISA, and the IGF-1 supplement treatment reduced the acute brain injury and mortality of aging rats after ICH, which may partly mimic the therapeutic effects of young plasma. Our research data showed that young plasma and its circulating ‘youth factors’, such as IGF-1, could be considered as novel therapeutic strategies for the treatment of ICH in aging patients.

## Materials and methods

### Rats and mice

Young Sprague–Dawley rats (3 months old, 300–350 g) and adult rats (10–11 months old) were obtained from the Animal Center of Daping Hospital, The Army Medical University (The Third Military Medical University, Chongqing, China) and maintained in specific pathogen free (SPF) conditions in our Experimental Animal Center. Aging rats were purchased at 12–13 months old and maintained in our animal center for an additional 10 months (weight 500–650 g). Adult rats (13 months old) weighing between 450 and 600 g and aging rats were used for our experiments. Furthermore, young C57BL/6 male mice (3 months old, 25–30 g) and adult mice (12–13 months old) were purchased from the Animal Center of Daping Hospital and maintained in our Experimental Animal Center. The old mice were purchased at 12–13 months old and were maintained in our animal center until they were 20–21 months old and weighed 30–35 g. Rats and mice were randomly divided into groups and investigators were blinded to the group information. Our animal study protocols were approved by and conducted in accordance with the Animal Ethics Committee of the Army Medical University (Third Military Medical University, Chongqing, China), and ARRIVE (Animal Research: Reporting *In Vivo* Experiments).

### Acute brain injury animal model induced by ICH

We created the rat ICH model by inducing an ICH in the striatum (caudate + putamen) according to a previous study [16]. In brief, the rats were anesthetized with isoflurane at 3.5% for induction and 1.5% for maintenance before being immobilized on a stereotaxic apparatus (RWD Life Science Co., Shenzhen, China). A 1-mm diameter burr hole was drilled 0.2 mm anterior and 3.5 mm lateral to the bregma. A total of 75-μl autologous blood was injected into the striatum after attaching a Hamilton needle to the micropump (RWD Life Science Co.) located at 5.5 mm ventral to the skull surface. The mouse ICH model was established according to our previous study [17], which is similar to the rat model. A total of 20 μl autologous blood was injected into the striatum (bregma 0: 0.8 mm anterior, 2 mm left lateral, and 3.5 mm deep) for mice.

### Parabiosis model

Male mice of similar weight and size were first paired for 2–3 weeks before the parabiotic surgery to ensure minimal variation and compatibility with our previous study [17]. After 2–3 weeks, we performed the old–young heterochronic and old–old isochronic parabiotic surgeries to investigate the effect of periphery blood on the treatment of ICH-induced acute brain injury. The parabiotic mice ICH model was conducted 50 days after parabiotic surgery and 5 days recovery after the separating surgery because the aging-related phenotypes would require 5 weeks for improvement after parabiotic surgery, according to a previous study [12].

### Rat plasma collection and injection into experimental animals

Blood samples were collected from the young (3–4 months old) and aging (22–23 months old) rats by removal of the eye using ethylenediaminetetraacetic acid (EDTA)-coated tubes. The samples were centrifuged at 1000×***g*** for 20 min to extract the plasma. Next, the plasma samples were dialyzed using 3.5 kDa D-tube dialyzers (Millipore, Billerica, MA, U.S.A.) in PBS to remove the EDTA before being stored at −80°C. For our experiments, aging ICH rats were systemically treated with 500 µl of plasma from young or old rats and the same volume of 0.9% saline as control by a single tail vein injection 30 min after the ICH surgery.

### Analysis of brain water content (BWC)

Three days after the ICH procedure, the rats were re-anesthetized and the ipsilateral brain tissues were resected for analysis of the brain water content (BWC) levels according to our previous study [17]. First, the resected brain tissues were wiped with filter paper to remove any excess water on the surface before being weighed. Next, the brain tissues were dried at 100°C for 24 h to determine the dry weight, and the BWC was calculated using the following formula: (%) = (wet weight − dry weight)/wet weight × 100%.

### Assessment of neurological deficient score (NDS)

On days 1, 3, 5, and 7 after ICH surgery with or without treatments, the mouse [17] and rat [18] neurological deficient score (NDS) were assessed, along with neurological deficiency scoring, by three trained investigators who were blinded to the experimental data. The NDS values were averaged and summarized as mean ± SD.

### Hematoxylin and eosin (H&E) staining

Brain tissues were resected from euthanized mice and rats, three sections from each animal were selected at random and fixed in a 10% neutral-buffered formalin solution. Next, the tissues were dehydrated in ethanol solutions and embedded in paraffin for sectioning with a rotary microtome at 5 µm thickness. Last, the sections were subsequently stained with hematoxylin and eosin (H&E) and visualized under a Leica microscope.

### Fluoro-Jade B (FJB) staining

We utilized the Fluoro-Jade B (FJB) stain to assess degenerative neurons according to our previous study [19]. Briefly, brain tissues were dehydrated in 15–30% sucrose solutions and cut into 25 µm thick sections. Next, three sections from each animal were selected at random and these sections were immersed in 1% sodium hydroxide in 80% alcohol for 10 min, followed by 70% alcohol for 2 min, and distilled water for 5 min. The sections were incubated in 0.06% potassium permanganate for 10 min at room temperature and then washed with distilled water. After washing, the sections were incubated in 0.01% FJB solution (Millipore, MA, U.S.A.) for 30 min and washed with distilled water. Next, the sections were dehydrated in gradient alcohol solutions and cleared in xylene and covered with coverslips in DPX (Sigma-Aldrich, St. Louis, MO, U.S.A.), a non-aqueous, non-fluorescent, plastic mounting medium. The FJB-stained tissue sections were reviewed and quantified based on the number of cells in each standardized microscopic field independently by three researchers with ImageJ (version 1.46J, National Institutes of Health). Positively stained cells were counted around the hematoma, and the number of positive cells in each field (magnified 100 times) was the averaged values of three regions of interest.

### Terminal deoxynucleotidyl transferase dUTP nick end labeling (TUNEL) and Nissl staining

A commercial *In Situ* Cell Death Detection kit (Roche, Basel, Switzerland) was used for terminal deoxynucleotidyl transferase dUTP nick end labeling (TUNEL) staining according to the manufacturer’s instructions. Briefly, 50 μl TUNEL reaction mixture was added to the three brain sections selected at random from each animal and incubated at 37°C for 1 h. Next, the slides were washed three times with Tris-buffered saline (TBS), while 50 μl of the marking solution was added to the negative control sections. After washing, the sections were incubated with 50–100 μl 3,3′-diaminobenzidine (DAB) solution or POD substitute solution for 10 min, and then dehydrated with gradient alcohol solutions, cleared in xylene, and covered with coverslips using neutral gum (Sigma-Aldrich). For the Nissl staining, tissue sections were stained with thionin (Sigma-Aldrich), dehydrated in alcohol, cleared in xylenes, and sealed with coverslips. The perihematomal and contralateral brain tissues were analyzed. The TUNEL and Nissl-stained tissue sections were analyzed under a Lycra microscope. For quantification of TUNEL and Nissl staining results, the number of cells in each standardized microscopic field was evaluated independently by three researchers using ImageJ (version 1.46J, National Institutes of Health). Positively stained cells were counted around the hematoma, and the number of positive cells in each field (magnified 100 times) was the averaged values of three regions of interest.

### Human plasma collection

Normal plasma was obtained from the Health Examinations Center of Xinqiao Hospital, Army Medical University (Third Military Medical University). The plasma donations and experiments were reviewed and approved by the Ethics Committee of Xinqiao Hospital, Army Medical University (Third Military Medical University). Written informed consent was obtained before the acquisition of plasma samples from all participants. Samples were confirmed to be normal based on the following assessments: blood pressure, serum glucose, serum lipid, leukocyte, and hemoglobin (Table 1). Additionally, we excluded participants who had chronic diseases, such as hepatitis, heart disease, viral and bacterial infection within 6 months, and immune system-related diseases. A total of 118 participants were used in the present study and divided into three groups (Young, Middle, and Old) by age. Blood (5 ml) was drawn from the antecubital vein and collected in EDTA tubes. Blood samples were centrifuged at 1000 ×***g*** for 20 min. The plasma samples were stored at −80°C until further analysis. From each age group, including young (21–30 years old), middle (41–50 years old), and old (≥ 60 years old), three to five samples were mixed as one to be measured by LC–MS/MS and ELISA. In addition, the research has been carried out in accordance with the World Medical Association Declaration of Helsinki.

**Table 1:** General and clinical characteristics of human participants

Characteristics	Young (*n*=36)	Middle (*n*=52)	Old (*n*=30)
Age (years), mean ± SD	26.5 ± 2.69	45.3 ± 2.96	66.1 ± 6.85
Male sex, n (%)	18 (50.00%)	22 (42.31%)	17 (56.67%)
Systolic pressure, mean ± SD	118.9 ± 8.79	124.0 ± 7.89	129.1± 9.69
Diastolic pressure, mean ± SD	74.7 ± 8.01	77.8 ± 5.90	73.2 ± 7.39
Triglyceride, mean ± SD	0.95 ± 0.39	1.08 ± 0.64	1.14 ± 0.37
Blood glucose, mean ± SD	5.03 ± 0.33	5.30 ± 0.41	5.59 ± 0.46
Hemoglobin, mean ± SD	141.97 ± 15.93	136.87 ± 13.40	133.40 ± 14.58
Leukocyte, mean ± SD	6.20 ± 1.34	5.45 ± 0.99	5.80 ± 1.63
Lymphocytes percentage, mean ± SD	33.45 ± 7.15	31.45 ± 5.70	28.99 ± 5.32
Neutrophil percentage, mean ± SD	57.69 ± 7.56	60.18 ± 6.69	60.52 ± 11.53

### Tandem mass tag (TMT) labeling and HPLC fractionation

The peptide was desalted using the Strata X C18 SPE column (Phenomenex, Torrance, CA, U.S.A.) and vacuum-dried. The peptide was reconstituted in 0.5 M tetraethylammonium bromide (TEAB) and processed according to the manufacturer’s protocol for the tandem mass tag (TMT) kit/iTRAQ kit. Briefly, 30 samples were divided into five groups. Each group was labeled with one unit of six-plex TMT reagent kits. The TMT reagents were thawed and reconstituted in acetonitrile. The peptide mixtures were then incubated for 2 h at room temperature and pooled, desalted, and dried by vacuum centrifugation. The labeled peptides were fractionated by high pH reverse-phase HPLC using Agilent 300Extend C18 column (5 μm particles, 4.6 mm ID, 250 mm length). Next, the peptides were first separated using a gradient of 8–32% acetonitrile (pH 9.0) over 60 min into 60 fractions. Then, the peptides were combined into 18 fractions and dried by vacuum centrifugation.

### LC–MS/MS analysis

Peptides were dissolved in 0.1% formic acid (solvent A), directly loaded into a home-made reversed-phase analytical column (15 cm length, 75 μm i.d.). The gradient was comprised of an increase from 6 to 20% of solvent B (0.1% formic acid in 98% acetonitrile) over 60 min, 20 to 30% in 22 min, climbing to 80% in 4 min, and then holding at 80% for the last 4 min, all at a constant flow rate of 350 nl/min on an EASY-nLC 1000 UPLC system.

The peptides were subjected to NSI source followed by MS/MS in Orbitrap fusion lumos (Thermo Fisher) coupled online to the UPLC. The electrospray voltage applied was 2.4 kV. The m/z scan range was 350–1550 for the full scan, and intact peptides were detected in the Orbitrap at a resolution of 60000. Peptides were then selected for MS/MS using the NCE setting as 28 and the fragments were detected in the Orbitrap at a resolution of 15000. A data-dependent procedure that alternated between one MS scan followed by 20 MS/MS scans with a 15.0 s dynamic exclusion. Automatic gain control (AGC) was set to 5E4, and the fixed first mass was set to 100 m/z.

### Data analysis

The resulting MS/MS data were processed using the Maxquant search engine (v.1.5.2.8). Tandem mass spectra were searched against UniProt Human database concatenated with reverse decoy database. Trypsin/P was specified as the cleavage enzyme allowing up to two missing cleavages. The mass tolerance for precursor ions was set to 20 ppm in first search and 5 ppm in the main search, and the mass tolerance for fragment ions was set as 0.02 Da. Carbamidomethyl on Cys was specified as the fixed modification and oxidation on Met was specified as the variable modification. The FDR was adjusted to less than 1%, and the minimum score for peptides was set to greater than 40.

### Real-time PCR

Total RNA was extracted by using Trizol reagent (Thermo Fisher Scientific), and cDNA was reverse transcribed in a 20 ml reaction volume using a High Capacity cDNA Reverse Transcription Kit (Thermo Fisher Scientific). Real-time PCR was performed using primers for *IGF-1* gene coding the following: F: GCTGAAGCCGTTCATTTAGC, R: GAGGAGGCCAAATTCAACAA. PCR was conducted in accordance with the Maxima SYBR Green/ROX qPCR Master Mix manufacturer’s instructions (Thermo Fisher Scientific). The thermal cycling conditions were as follows: 2 min at 50°C and 10 min at 95°C followed by 40 cycles at 95°C for 15 s and 1 min at 60°C. The experimental data were analyzed by using the ^ΔΔ^*C_t_* method. The mRNA expression of genes of interest was normalized to the glyceraldehyde 3-phosphate dehydrogenase (GAPDH) mRNA level.

### Plasma IGF-1 measurements

Plasma IGF-1 levels were determined using a commercial IGF-1 detection kit according to the manufacturer’s recommendation (human and rat IGF-1: R&D system, Minneapolis, MN, U.S.A.).

### Injection of IGF-1 into experimental animals

Recombinant rat IGF-1 (R&D system, Minneapolis, MN, U.S.A.; 10 μg/ml) and a vehicle (PBS) were administered via intraperitoneal injection 30 min after the ICH surgery to assess the effects of IGF-1 on the regulation of ICH-induced acute brain injury.

### Statistical analysis

Analyses were performed with SPSS 13.0 software, and all values are presented as mean ± SD or percentage. The Student’s *t*-test for independent samples was used to compare two groups, and multiple groups were compared by using 1-way ANOVA followed by the Student–Newman–Keuls *post hoc* test. Two-way ANOVAs with repeated measures were performed when appropriate to compare repeated measured data. *P<0.05, **P<0.01, and ***P<0.001 presented different levels of statistical significance.

## Results

### Young plasma reduces acute brain injury in aging ICH rodents

**Figure 1:**
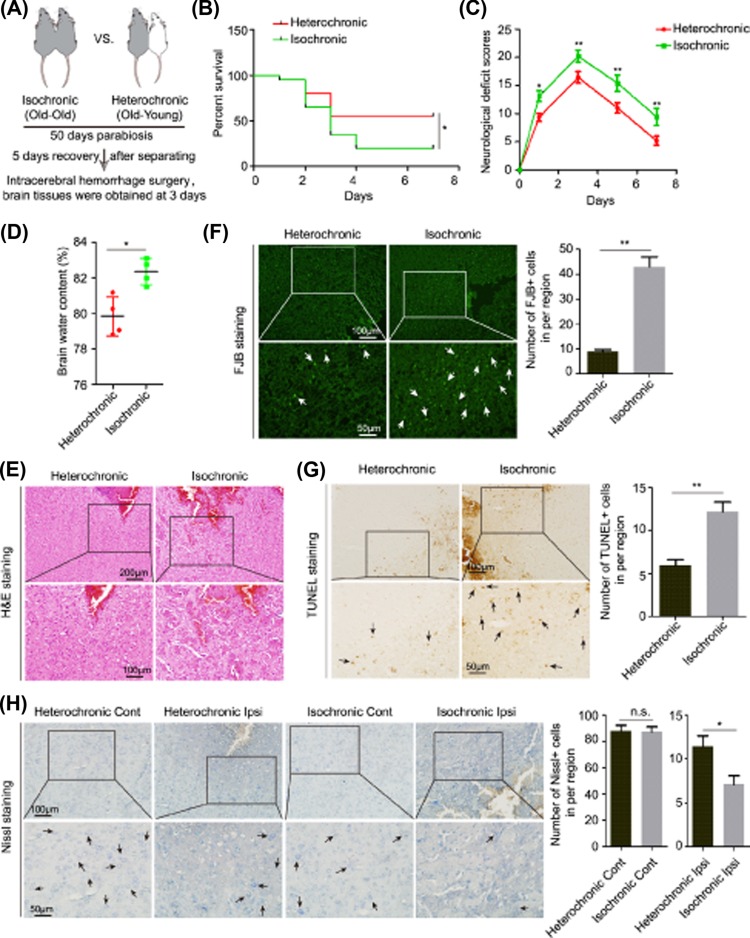
Favorable indices of neurological injuries in heterochronic parabiont mice compared with isochronic parabiont mice after ICH surgery (**A**) Establishment of parabiosis and parabiont ICH mouse models. (**B**) Mortality rates of heterochronic and isochronic parabiont ICH mice (*n*=20). (**C**) NDS of heterochronic and isochronic parabiont ICH mice (*n*=4–11 for each group). (**D**) BWC levels in the perihematomal brain tissues of heterochronic and isochronic parabiont mice 3 days after ICH surgery (*n*=4). (**E**) H&E staining of perihematomal brain tissues derived from heterochronic and isochronic parabiont mice 3 days after ICH. Scale bars, 200 μm on the top and 100 μm on the bottom. (**F**) FJB staining and quantification of degenerative neural cells in perihematomal brain tissues derived from heterochronic and isochronic parabiont mice 3 days after ICH. Scale bars, 100 μm on the top and 50 μm on the bottom. (**G**) TUNEL assay and quantification of apoptotic neural cells in perihematomal brain tissues derived from heterochronic and isochronic parabiont mice 3 days after ICH. Scale bars, 100 μm on the top and 50 μm on the bottom. (**H**) Nissl staining and quantification of Nissl bodies in perihematomal brain tissues derived from heterochronic and isochronic parabiont mice 3 days after ICH. Scale bars, 100 μm on the top and 50 μm on the bottom. The bar graphs show the mean ± SD. *P*-values were determined by the Student’s *t* test. *P<0.05 and **P<0.01, respectively. Abbreviation: NDS, neurological deficit score

We explored whether blood or plasma from young rodents were able to reduce the severity of acute brain injury in aging ICH rodents. The old (22 months) isochronic and heterochronic parabiont mice were produced and about 50 days later and 5 days recovery of separating surgery, ICH surgery was conducted (Figure 1A); while all the parabiont rats in the present study were dead at about 3–5 days after symbiotic surgery with unknown reasons, although we have used the antibacterial agents. Therefore, we only observed the data of mice and found a significant reduction in ICH-induced acute brain damage, along with an improvement of mortality and neurological function, in the heterochronic parabionts when compared with the isochronic parabionts (Figure 1B–H).

**Figure 2:**
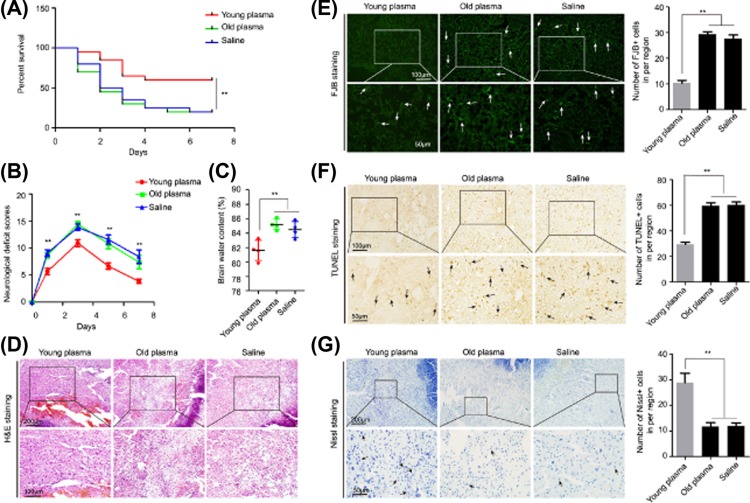
Treatment of aging ICH rats with young plasma reduces ICH-induced acute brain injury (**A**) The mortality rates of aging ICH rats after treatment with young or old plasma (*n*=20). (**B**) The NDS in aging ICH rats after treatment with young or old plasma or saline (*n*=4–12 for each group). (**C**) BWC in the perihematomal brain tissues of aging ICH rats 3 days after being treated with young or old plasma or saline (*n*=4). (**D**) H&E staining of perihematomal brain tissues obtained from aging ICH rats 3 days after being treated with young or old plasma or saline (Scale bars, 200 μm on the top and 100 μm on the bottom). (**E**) FJB staining and quantification of degenerative neural cells in the perihematomal brain tissues derived from aging ICH rats 3 days after being treated with young or old plasma or saline (Scale bars, 100 μm on the top and 50 μm on the bottom). (**F**) TUNEL assay and quantification of apoptotic neurons in perihematomal brain tissues derived from aging ICH rats 3 days after being treated with young or old plasma or saline (Scale bars, 100 μm on the top and 50 μm on the bottom). (**G**) Nissl staining and quantification of Nissl bodies in perihematomal brain tissues obtained from aging ICH rats 3 days after being treated with young or old plasma or saline (Scale bars, 200 μm on the top and 50 μm on the bottom). The bar graph data are expressed as the mean ± SD. *P*-values were determined using the Student’s *t*-test. *P<0.05 and **P<0.01, respectively.

Then, we investigated whether the young plasma could also have potential therapeutic effects on acute brain injury in aging ICH rats. Five hundred microliter young plasma was injected once into aging ICH rats via tail vein 30 min after surgery. The results showed that the mortality rates (Figure 2A) and neurological deficit scores (NDS) (Figure 2B) in young plasma-treated aging ICH rat group were significantly reduced when compared with treatment with old plasma or saline. In addition, we found that treatment with young plasma significantly reduced the BWC (a measurement of brain damages) of perihematomal brain tissues (Figure 2C). The H&E staining showed that the severity of edema, the degree of necrosis, and inflammatory cell infiltration around the hematoma site were significantly alleviated in the young plasma treatment group compared with the other two groups (Figure 2D). Furthermore, the numbers of degenerative and apoptotic neural cells in aging ICH rats showed by FJB (Figure 2E) and TUNEL (Figure 2F) staining, respectively, were significantly decreased in young plasma-treated aging ICH rats compared with that treated with old plasma or saline; in contrast, the number of surviving neurons evidenced by Nissl staining in perihematomal brain tissues of aging ICH rats treated with young plasma were significantly increased (Figure 2G). These data indicated that young plasma treatment plays markedly therapeutic roles in the reduction of acute brain injury in aging ICH rodents.

### Plasma IGF-1 level was markedly decreased with age both in human and rats

**Figure 3:**
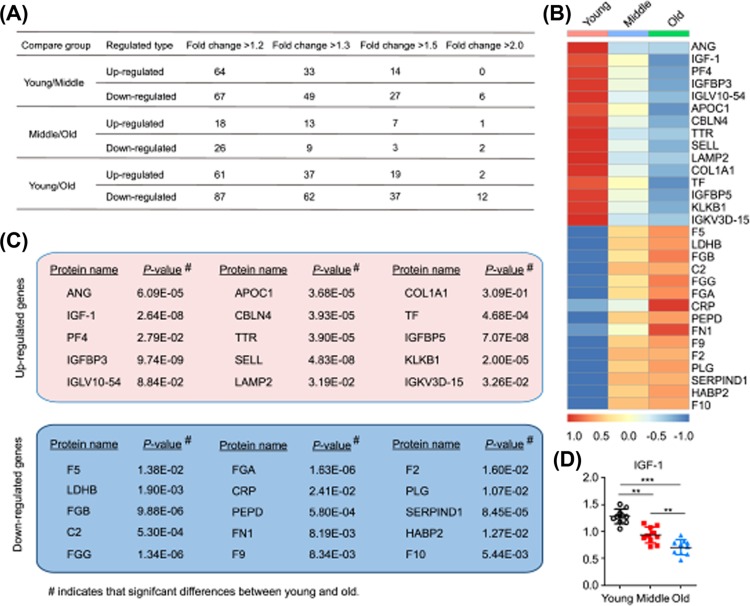
The potential neuroprotective circulating factors were screened using LC–MS/MS analysis (**A**) Differentially expressed protein summary (filtered with threshold value of expression fold change and *P*-value <0.05). (**B**) Heatmap shows the representative circulating factors significantly elevated and decreased in human plasma. (**C**) Representative changed circulating proteins between young and old plasma. MetaCore was used to identify core proteins in each plasma data set. (**D**) IGF-1 level changes in human plasma across different age groups detected by LC–MS/MS analysis. **P<0.01 and ***P<0.001, respectively.

**Figure 4:**
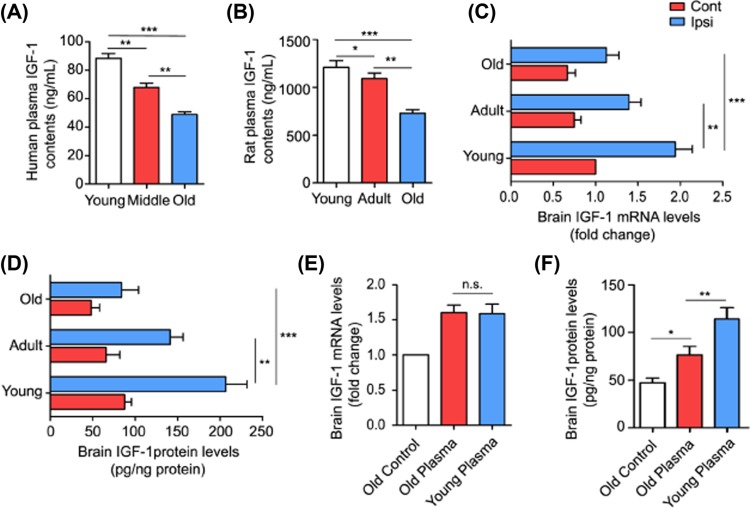
Changes in brain and plasma IGF-1 levels in rats across all age groups (**A, B**) IGF-1 levels in human (A) and rat (B) plasma in young, middle (adult), and aging rats detected by ELISA (*n*=4). (**C, D**) IGF-1 mRNA (C) and protein (D) levels in ipsilateral and contralateral brain tissues of young, adult, and aging ICH rats detected by RT-PCR and ELISA, respectively (*n*=4). (**E, F**) IGF-1 mRNA (E) and protein (F) levels in ipsilateral brain tissues of aging ICH rats treated with young or old rat plasma detected by RT-PCR and ELISA, respectively (*n*=4). The bar graphs show the mean ± SD. *P*-values were determined by the Student’s *t* test. *P<0.05, **P<0.01, and ***P 0.001, respectively, n.s. indicates no significance.

Given that circulating factors are key in reversing age-related brain functions [7,10,13,14,20]. Therefore, to determine the changes in circulating factors with age and explore their potential for use as therapeutic approaches to acute brain injury in aging ICH rodents, we first performed the proteomics analysis to identify proteins in plasma from young (20–30 years old), middle (40–50 years old), and older (>60 years old) healthy humans (Table 1) and more than 1000 proteins of diverse functional classes were detected, among them 845 proteins can be quantified (Supplementary Table S1). Proteomics analysis further showed that many circulating proteins were significantly up-regulated or down-regulated in plasma range from young to old participates (Figure 3A–C); among them, the plasma IGF-1 levels gradually and markedly decreased with age (Figure 3D). Moreover, we found that IGF-1 was higher in the young plasma compared with the middle and old plasma both in health human (Figure 4A) and normal rats (Figure 4B) using an ELISA. Before investigating whether blood-borne IGF-1 could reduce brain injury in aging ICH rats, we assessed IGF-1 levels in the perihematomal brain tissues of rats. The qPCR data showed that *IGF-1* mRNA levels in normal brain tissues gradually decreased from young to old rats (Figure 4C), as well as the IGF-1 protein levels detected by ELISA (Figure 4D). 3 days after ICH, the increasing folds of *IGF-1* mRNA level in the perihematomal brain tissues in young rats was higher than that in adult and old rats (Figure 4C), and the same changes in IGF-1 protein level were also obtained after ICH (Figure 4D). Meanwhile, the young and old plasma treatment did not significantly increase the level of brain *IGF-1* mRNA in aging ICH rat brains (Figure 4E), but the level of IGF-1 protein in the perihematomal brain tissues was increased in the young plasma treatment group (Figure 4F). These results suggested that the blood-borne IGF-1 could enter the brain tissue crossing the blood–brain barrier (BBB) [21,22], and may exert neuroprotective roles in aging ICH rats.

### IGF-1 protects against acute brain injury in aging ICH rats

**Figure 5:**
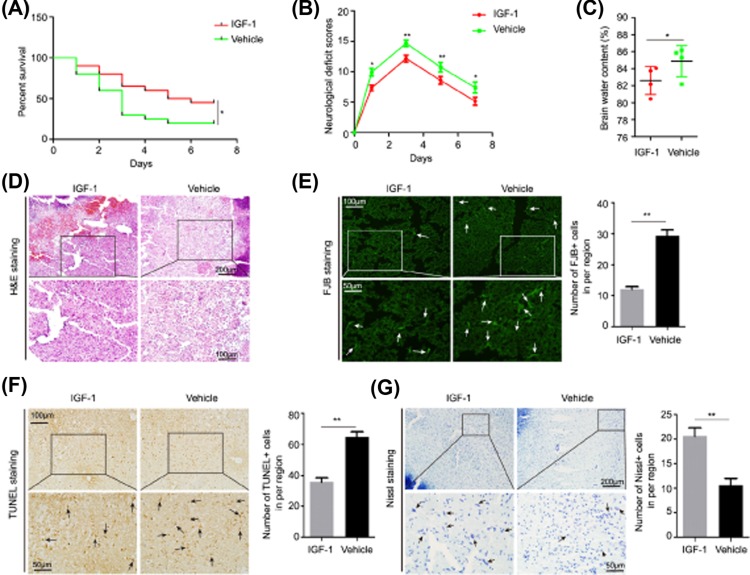
IGF-1 reduction of acute brain injury in aging ICH rats (**A**) The mortality of aging ICH rats after treated with the IGF-1 protein or vehicle (*n*=20). (**B**) The NDS of aging ICH rats after being treated with the IGF-1 protein or vehicle (*n*=4–9). (**C**) Level of the BWCs in the perihematomal brain tissues in aging ICH rats 3 days after treatment with the IGF-1 protein or vehicle (*n*=4). (**D**) H&E staining of the perihematomal brain tissues derived from aging ICH rats 3 days after treatment with the IGF-1 protein or vehicle. Scale bars, 200 μm on the top and 100 μm on the bottom. (**E**) FJB staining and quantification of degenerative neural cells in the perihematomal brain tissues derived from aging ICH rats 3 days after treatment with the IGF-1 protein or vehicle. Scale bars, 100 μm on the top and 50 μm on the bottom. (**F**) TUNEL assay and quantification of apoptotic neural cells in perihematomal brain tissues derived from aging ICH rats 3 days after treatment with the IGF-1 protein or vehicle. Scale bars, 100 μm on the top and 50 μm on the bottom. (**G**) Nissl staining and quantification of Nissl bodies in perihematomal brain tissues derived from aging ICH rats 3 days after treatment with the IGF-1 protein or vehicle. Scale bars, 200 μm on the top and 50 μm on the bottom. The bar graphs show the mean ± SD. *P*-values were determined by the Student’s *t* test. *P<0.05, **P<0.01, and ***P<0.001, respectively. n.s. indicates no significance.

To further confirm this hypothesis, we administered the IGF-1 protein to the old ICH rats via intraperitoneal injection 30 min after ICH surgery and found that IGF-1 treatment significantly reduced the mortality (Figure 5A) rates and NDS (Figure 5B) of aging ICH rats, which were closely correlated with the reduction of BMC (Figure 5C), edema severity, necrosis degree, inflammatory cell infiltration around the hematoma site (Figure 5D), the numbers of degenerative and apoptotic neural cells (Figure 5E,F), and the increase in the number of surviving neurons (Figure 5G). These results suggested that the circulating IGF-1 could enter the brain and exert neuroprotective effects on reducing acute brain injury in aging ICH rats.

## Discussion

In the current study, we revealed the therapeutic effects of young plasma treatment on reducing acute brain injury in aging ICH rats. The acute brain injury in aging ICH rats was significantly reduced when received the treatment of young plasma, which contains many functional circulating factors that have been shown to reverse the brain biofunctions in aging [10,12–14,20]. Among these functional circulating factors, using the proteomics analysis we screened out that plasma IGF-1 were markedly decreased with age, which was further confirmed in rats. In addition, the supplement treatment of IGF-1, which likely replaced the IGF-1 loss in old plasma, resulted in reduced acute brain injury in the aging ICH rats. Our study showed that young plasma may be considered as a potential clinical application for treatment of acute stroke in elderly patients.

Aging can influence the systemic responses to chronic brain dysfunction and acute brain injury [14], and young plasma has been shown to reverse chronic brain dysfunction in aging mice [10,11]. Clinical trials have been conducted to investigate its role in human cognitive disorders [6,7,10,23]. Many of these previous studies showed the protective effects of young plasma on aging were chronic dysfunction, and our study found that the young plasma could also protect against the acute injury in aging. Among these indices, the very high mortality of aging ICH rats may be correlated with the advanced age (22–23 months old) and larger autologous blood volume (75 μl), and the two factors added-up might be deadly to the rats. While, treating them with young plasma significantly reduced their mortality in the present study. These results strongly suggest that young plasma also have protective effects on aging-related acute brain injury. Although the injection time of young plasma might be so early for the vast majority of clinical subjects and lacking of mechanistic insights into how young plasma would result in better function after ICH in our study, which may not influence the young plasma treatment as a novel therapeutic direction for the clinical patients with ICH. Besides, there is no reference dose of young rat plasma to treat diseases, so we just tried the 500 μl volume of young rat plasma and did not investigate the other dosages. Although effective of 500 μl young rat plasma administrated at as early as 30 min after ICH in the present study, the other therapeutic time and best dosages and how the young plasma changes the brain tissues after injury still deserve to be investigated in future studies.

Given that young plasma has complex factors that may be beneficial or harmful, finding out the beneficial circulating factors may also be helpful for improving brain functions. Therefore, studies have revealed some circulating factors, such as myostatin [24], IGF-1 [25], IGF-2 [26], and TIMP2 [14], in the young plasma from animals and human, and also known as ‘youth factors’, because which have been shown to improve organ or tissue dysfunctions in aging rodents. However, it is unknown what kind of circulating factors in the young plasma could protect against acute brain injury caused by ICH in aging. Therefore, we identified changes in plasma proteins in healthy human of different age groups using proteomics analysis. More than 1000 plasma proteins were identified, and the IGF-1 level was markedly decreased with age both in human and rats. Many studies have revealed the IGF-1 has important roles in regulating brain injury [27–29] and aging [30,31], some studies conclude beneficial roles [21,22,32], while some thought IGF-1 confers impairment roles [33–35]. In our study, consisting with some of these previous studies [21,22], we found that circulating IGF-1 in the young plasma could enter the brain and significantly protect against acute brain injury. However, the exact reasons of controversial conclusion of IGF-1 on regulating brain injury and aging were unknown. Besides, several other plasma proteins were also found to be significantly changed with age in our study, such as APOC1, which may also exert similar neuroprotective roles and deserves further investigating. Altogether, these findings suggest that the circulating ‘youth factors’, like IGF-1, may act as the critical regulator in the systemic proteome and perhaps as the systemic CNS communication to reduce the aging-related acute brain injury after stroke.

## Conclusion

In summary, our study provided a novel therapeutic strategy for treatment of acute brain injury caused by ICH in aging, and future studies are needed to further assess whether such treatment could reduce the severity of acute stroke in aging patients. Collectively, identification of these rejuvenating factor or factors could be exploited to develop new drugs to treat brain injuries in diseases of pathologic aging.

## Clinical perspectives

- Aging has been shown to contribute to both the declined biofunctions of aging brain and aggravation of acute brain damage and the former could be reversed by young plasma; however, whether young plasma treatment may also reduce the acute brain damage induced by ICH remains explore.
- Young plasma treatment significantly reduced the improved outcomes of aging ICH rodents, which might be due to the neuroprotective circulating factors from young plasma, such as IGF-1.
- These results indicated that young plasma may be considered as a novel therapeutic approach for the clinical treatment of aging-related acute brain injury.

## Supporting information

**Supplementary Table S1:** 

## Abbreviations

BBB: blood–brain barrier
BMC: brain water content
EDTA: ethylenediaminetetraacetic acid
FJB: Fluoro-Jade B
H&E: hematoxylin and eosin
HPLC: high-performance liquid chromatography
ICH: intracerebral hemorrhage
IGF-1: insulin-like growth factor 1
NDS: neurological deficient score
PBS: phosphate buffered saline
TMT: tandem mass tag
TUNEL: terminal deoxynucleotidyl transferase dUTP nick end labeling
